# High Fat Diet Induces Kidney Injury *via* Stimulating Wnt/β-Catenin Signaling

**DOI:** 10.3389/fmed.2022.851618

**Published:** 2022-04-07

**Authors:** Ying Yu, Hongyan Mo, Hui Zhuo, Chen Yu, Youhua Liu

**Affiliations:** ^1^Department of Pathology, University of Pittsburgh School of Medicine, Pittsburgh, PA, United States; ^2^Department of Nephrology, Tongji Hospital, School of Medicine, Tongji University, Shanghai, China; ^3^State Key Laboratory of Organ Failure Research, Division of Nephrology, National Clinical Research Center of Kidney Disease, Nanfang Hospital, Southern Medical University, Guangzhou, China

**Keywords:** high fat, obesity, Wnt/β-catenin, kidney injury, podocyte, ICG-001

## Abstract

High fat diet could cause kidney injury, and the underlying mechanism remains incompletely understood. In this study, we investigated the role of Wnt signaling in this process. Mice were fed with high-fat diet *in vivo*, and podocytes were stimulated with palmitate *in vitro*. In mice fed with high-fat diet, renal function was impaired, accompanied by induction of various proinflammatory cytokines and proteinuria. Renal expression of Wnt ligands was also significantly induced, with Wnt1 and Wnt3a being the most pronounced, in high-fat diet mice, compared with normal diet controls. Intervention with ICG-001, a small molecule Wnt/β-catenin inhibitor, improved renal function, inhibited proinflammatory cytokines expression, reduced proteinuria and alleviated podocyte injury. In palmitate-treated podocytes, intracellular lipid deposition was increased, Wnt1 and Wnt3a expression was up-regulated, which was accompanied by an increased proinflammatory cytokines expression and podocyte injury. These lesions caused by palmitate were largely alleviated by ICG-001. Furthermore, ICG-001 also restored the expression of phosphorylated AMPK repressed by palmitate in podocytes or a high-fat diet in mice. These studies suggest that Wnt/β-catenin signaling is involved in the pathogenesis of high-fat diet-induced kidney injury. Targeting this signaling may be a potential therapeutic strategy for alleviating obesity-related nephropathy.

## Introduction

Over the past 30 years, the population of overweight or obese people has been growing worldwide (1). In recent years, more and more attention has been paid to obesity-associated kidney injury, which can occur early in obesity (2). Obese people have a higher risk of developing end-stage renal disease (ESRD) (3), especially when combined with diabetes and hypertension (4, 5). Obesity causes inflammatory responses and oxidative stress through hemodynamic alterations and activation of the renin-angiotensin system (RAS), leading to glomerulosclerosis and interstitial fibrosis, which ultimately impair renal structure and function (6–9). Although multiple factors are reported to contribute to obesity-induced nephropathy, the underlying mechanisms have not been fully understood.

Wnt/β-catenin is an evolutionarily conserved, key developmental signaling pathway implicated in both acute kidney injury and chronic kidney disease (10–12). Studies show that Wnt/β-catenin signaling plays a key role in the pathogenesis of podocyte dysfunction and kidney fibrosis (13, 14). The canonical Wnt signaling is initiated when a Wnt ligand binds to a member of the Frizzled (Fz) family of seven-transmembrane receptors and coreceptors LRP5/6, followed by stabilization of cytoplasmic β-catenin, which eventually translocates into the nucleus to activate target gene transcription.

Wnt proteins are lipid-modified, which makes them more stable and more conducive to play their roles (15, 16). This prompted us to investigate whether Wnt signaling plays a role in mediating high-fat diet-induced kidney injury. In the present study, we observed that a high-fat diet led to localized lipid deposition in kidney and renal structural and functional impairment, including increased inflammatory response, proteinuria and elevated serum creatinine, which can be ameliorated by ICG-001, a small molecule inhibitor of Wnt signaling. Our studies suggest a pivotal role of Wnt/β-catenin signaling in mediating obesity-induced nephropathy.

## Materials and Methods

### Animals

The 6-week-old CD1 mice were fed with normal diet (ND, 10% of total calories from fat) or high-fat diet (HFD, 60% of total calories from fat) (D12492; Research Diets Inc., New Brunswick, NJ) for 12 weeks. After 4 weeks of feeding ND or HFD, mice were intra-peritoneally injected daily with ICG-001 (kindly provided by Dr. M. Kahn, University of Southern California, Los Angeles, CA) at 5 mg/kg body wt. Mice were weighed and blood glucose measured weekly, and blood, urine, liver, and kidney tissue collected for the further experiments. Plasma levels of triglyceride (TG) and total cholesterol (TC) were determined using Thermo Infinity TG and cholesterol reagents (ThermoFisher Scientific, Middletown, VA).

### Cell Culture

The conditionally immortalized mouse podocyte cell line MPC-5 was provided by Peter Mundel (Massachusetts General Hospital, Boston, MA) and maintained as described previously (17). MPC-5 cells were cultured at 33°C in RPMI-1640 medium supplemented with 10% FBS and 10 units/ml mouse recombinant interferon-gamma (R&D Systems, Minneapolis, MN) to enhance the expression of a thermosensitive T antigen. For inducing differentiation, MPC-5 cells were cultured in non-permissive conditions at 37°C in the absence of interferon-gamma. Cells were stimulated by 0.1 or 0.2 mmol/L palmitate (P9767; Sigma, St Louis, MO) or 0.5% BSA (A0281; Sigma) and/or 5 μM ICG-001 for 24 h and then collected for further experiments.

### Determination of Serum Creatinine

Serum creatinine level was determined by using the QuantiChrom creatinine assay kit (BioAssay Systems, Hayward, CA), according to the instruction specified by the manufacturer.

### Immunofluorescent Staining

Kidney cryosections were fixed with 4% paraformalin for 15 min at room temperature and immersed in 0.2% Triton X-100 for 10 min. After washed by TBST, slides were blocked with 10% donkey serum for 1 h, then immunostained with primary antibodies against nephrin (20R-NP002; Fitzgerald Industries International, Acton, MA), WT1 (sc192; Santa Cruz Biotechnology, Dallas, TX) at 4°C overnight. After washing, slides were incubated with CY3-conjugated, affinity-purified secondary antibodies (Jackson ImmunoResearch Laboratories, West Grove, PA) for 1 h. Cell nuclei were stained using DAPI. Slides were observed using an Eclipse E600epi fluorescence microscope equipped with a digital camera (Nikon, Melville, NY).

### Immunohistochemical Staining

After deparaffinization and hydration, 4-μm paraffin-embedded sections were dewaxed and hydrated. Slides were then treated for antigen reparation and blocked. Specific antibodies against Wnt1 (ab15251; abcam), Wnt3a (SAB2108434; Sigma), β-catenin (610154; Becton Dickinson and Company) and CD45 (13917; Cell Signaling Technology, Danvers, MA), CD68 (26042; CST) were incubated at 4°C overnight, followed by incubation with biotin-labeled secondary antibodies and horseradish peroxidase-labeled tertiary antibodies (DAKO). Slides were stained, redyed with hematoxylin, dehydrated, and sealed in neutral rubber for the final light microscopy.

### Oil Red Staining

Kidney or liver cryosections or cell culture slides were fixed with 4% paraformaldehyde for 10 min, washed with PBS, incubated with 60% isopropanol for 2 min, stained with Oil Red O (Sigma) for 10–30 min, followed by washing with distilled water, re-staining with hematoxylin for 2 min, and washing and finally sealing with glycerol gelatin. The lipid droplets in the tissue or cells were orange-red, and the nuclei were blue.

### Real-Time PCR

Total RNA was isolated using TRIzol (Invitrogen) and reverse transcribed into cDNA using the 1st Strand cDNA Synthesis Kit (Promega). Quantitative, real-time RT-PCR was performed on ABI PRISM 7000 sequence detection system (Applied Biosystems, Foster City, CA) by standard protocols. The primers were listed in Supplementary Table 1.

### Western Blot Analysis

Kidney tissues or cells were lysed with RIPA buffer, and the supernatants were collected. Protein samples were separated using SDS-PAGE and transferred to polyvinylidene difluoride membranes. Membranes were blocked with 10% BSA for 1 h at room temperature and hybridized at 4°C overnight with specific antibodies: anti-Wnt1 (ab15251; abcam), anti-Wnt3a (SAB 2108434; Sigma), anti-β-catenin (610154; BD Biosciences, Frnklin Lakes, NJ), anti-α-tubulin (T9026; Sigma), anti-nephrin (20R-NP002; Fitzgerald), anti-WT1 (sc192; Santa Cruz Biotechnology), anti-AMPK (2532; CST), anti-p-AMPK (2535; CST). After incubation with the secondary antibody of the corresponding species origin, they were developed by the HRP-ECL luminescence method.

### Statistical Analyses

All data were expressed as mean ± SEM. Significant differences between the control and treated groups were determined by one-way ANOVA, followed by the Student-Newman-Keuls test using SPSS software (Chicago, IL). *P* < 0.05 was considered statistically significant.

## Results

### High-Fat Diet Induces Kidney Injury in Mice

**Figure 1:**
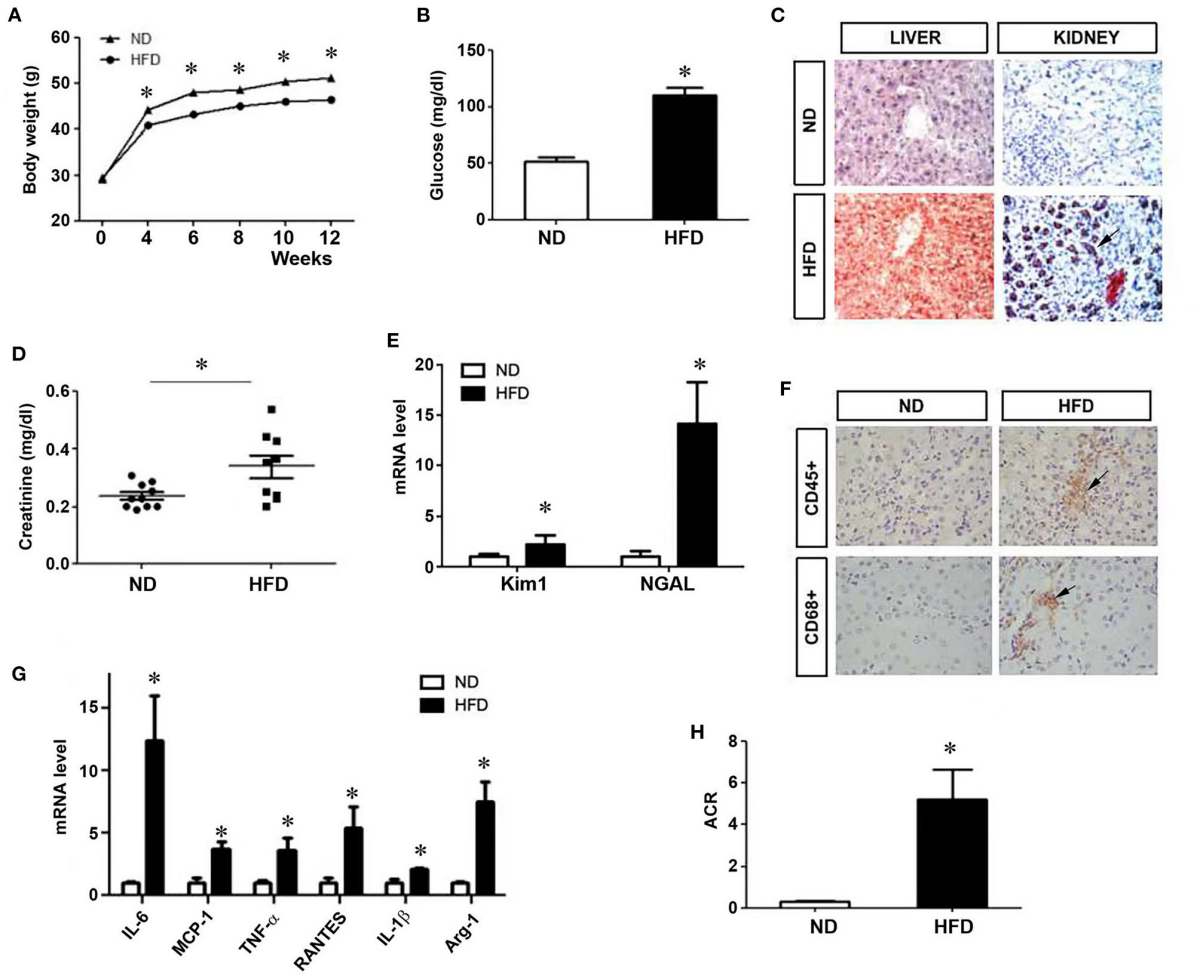
Kidney injury in mice on a high-fat diet. After mice were given a high-fat diet for 12 weeks, body weight **(A)** and blood glucose **(B)** were measured. **(C)** Liver and kidney tissues were subjected to oil red staining for lipid deposition. **(D)** Serum creatinine levels were measured. Renal expression of Kim1 and NGAL mRNA was assessed **(E)**. **(F)** Immunohistochemical staining for CD45+ and CD68+ cells in kidney tissues. **(G)** Quantitative real-time RT-PCR shows the relative abundances of proinflammatory cytokines as indicated. **(H)** Urinary albuminuria levels in different groups. Albumin to creatinine ratio (ACR) was presented. ND, Normal diet; HFD, high fat diet; *P < 0.05, ND vs. HFD, *n* = 6.

After 12 weeks of high-fat diet (HFD), body weight and blood glucose in mice increased clearly (Figures 1A,B), and there was substantial lipid deposition in the liver and kidney (Figure 1C). Seum creatinine level also increased (Figure 1D). Renal expression of kidney injury molecule-1 (kim-1) and neutrophil gelatinase-associated lipocalin (NGAL), two tubular damage markers, was upregulated (Figure 1E). Renal infiltration of CD68^+^ and CD45^+^ inflammatory cells was observed in the kidney of mice fed with HFD, compared to normal diet (ND) (Figure 1F). Consistently, renal expression of a variety of proinflammatory cytokines such as interleukin-6 (IL-6), monocyte chemoattractant protein-1 (MCP-1), tumor necrosis factor-α (TNF-α), regulated upon activation and normal T cell expressed and secreted (RANTES), IL-1β and arginase-1 (Arg-1) was also markedly induced (Figure 1G). Mice fed with HFD developed proteinuria (Figure 1H). These results indicate that HFD causes tubular injury, renal inflammation and proteinuria, leading to kidney dysfunction.

### Activation of Wnt/β-Catenin Signaling in HFD Mice

**Figure 2:**
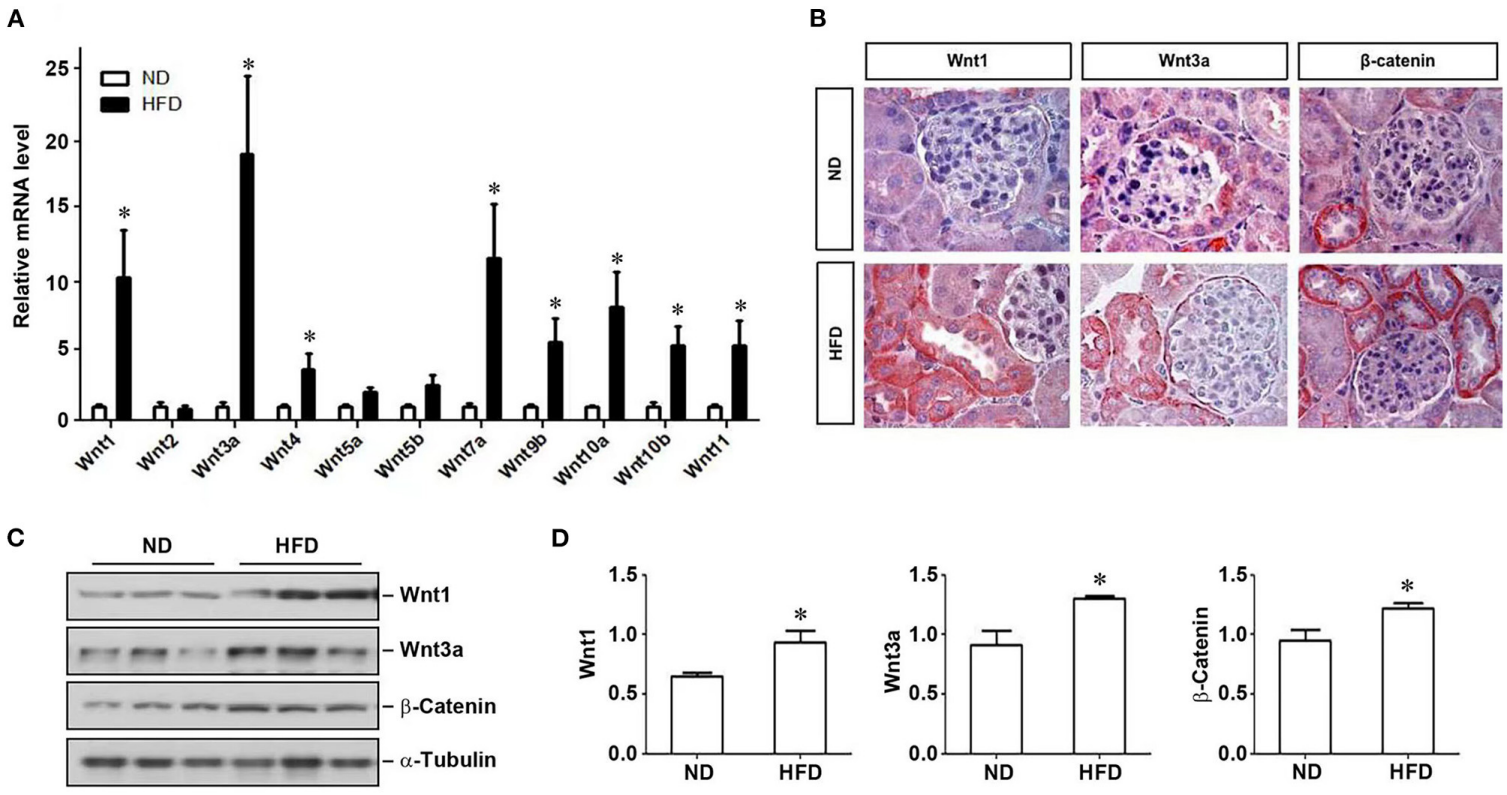
Activation of Wnt signaling pathway in mice on a high-fat diet. After 12 weeks of a high-fat diet in mice, kidney tissues were collected to detect the expression of Wnt family members by real-time RT-PCR **(A)**, the localization and expression of Wnt1, Wnt3a and β-catenin in kidney tissues by immunohistochemistry **(B)** and western blot **(C)**, respectively. **(D)** Quantitative determination of the expression levels of various proteins as indicated by densitometry and normalized with α-tubulin. ND, normal diet; HFD, high-fat diet; *P < 0.05, ND vs. HFD, *n* = 6.

We next examined the expression of multiple Wnt ligands in the kidney of mice fed with HFD. As shown in Figure 2A, many members of the Wnt family genes were induced in the kidney of mice fed with HFD, compared to ND, with Wnt1 and Wnt3a being the most significantly upregulated. Immunohistochemically staining confirmed that Wnt1, Wnt3a, and downstream β-catenin were significantly up-regulated in the kidney in mice fed with HFD (Figure 2B). Similarly, Western blotting also demonstrated that renal expression of Wnt1, Wnt3a and β-catenin proteins was upregulated (Figures 2C,D), indicating that a high-fat diet activates renal Wnt/β-catenin signaling.

### ICG-001 Ameliorates HFD-Induced Renal Injury in Mice

**Figure 3:**
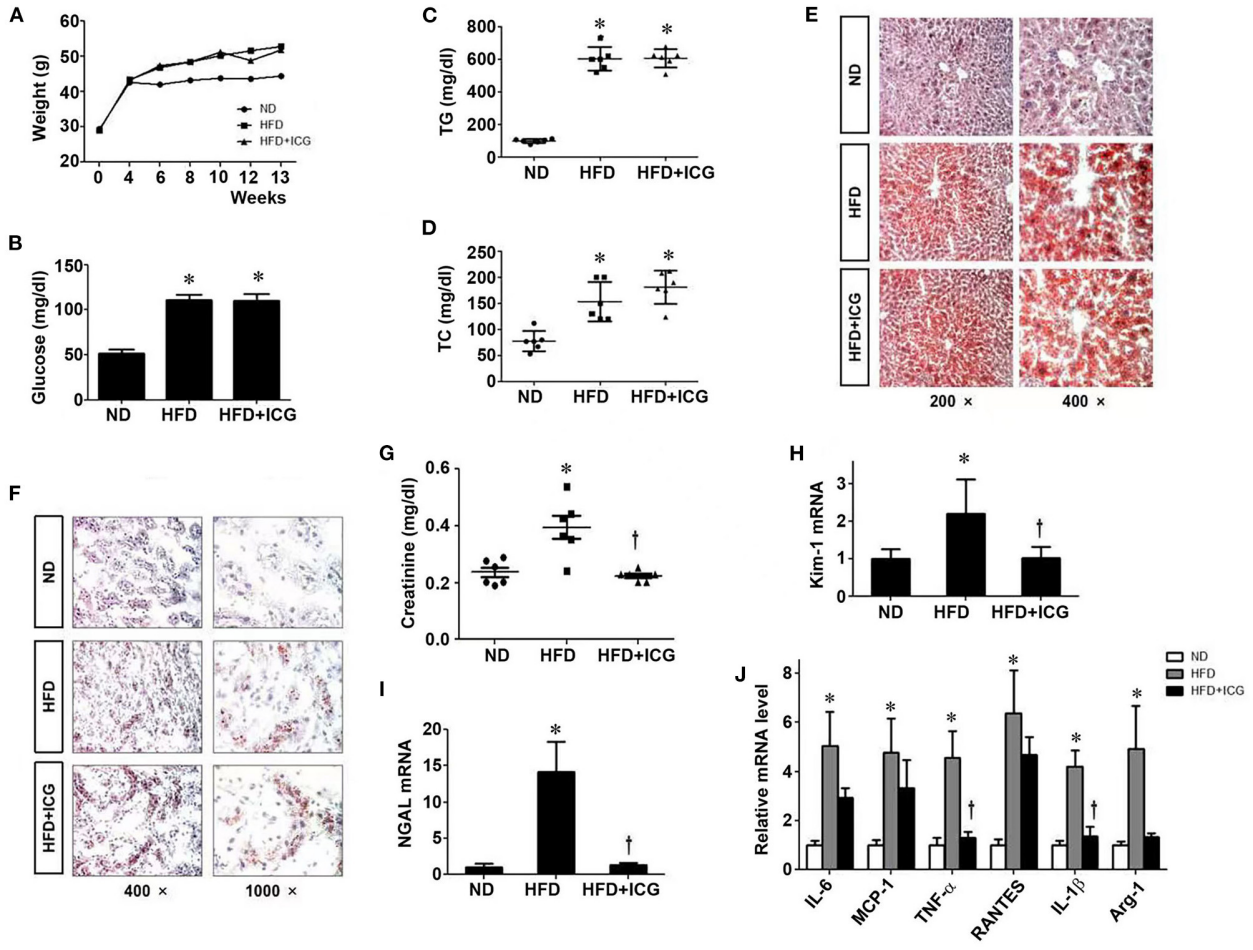
ICG-001 ameliorates kidney injury in mice on high-fat diet. After 4 weeks with high-fat diet, mice were injected with Wnt signaling inhibitor ICG-001 or vehicle (DMSO). Body weight **(A)**, blood glucose **(B)**, plasma triglyceride **(C)**, plasma total cholesterol **(D)** were measured at 9 weeks after ICG-001 treatment. **(E,F)** Oil-red staining for local lipid deposition in liver **(E)** and kidney **(F)**. **(G)** Serum creatinine levels. **(H,I)** Kidney injury was assessed by the markers of renal tubular damage, Kim-1 **(H)** and NGAL **(I)**. **(J)** Renal expression of various cytokines was assessed by real-time PCR. ND, normal die; HFD, high-fat diet; TG, triglyceride; TC, total cholesterol. *P < 0.05, ND vs. HFD, *P* < 0.05 HFD vs. HFD-ICG, *n* = 6.

Since HFD induces Wnt/β-catenin signaling in the kidney, we then wondered whether this signaling plays a role in mediating HFD-induced kidney injury. To this end, we treated HFD mice with ICG-001, a small molecule inhibitor of β-catenin signaling, starting at 4 weeks after HFD. As shown in Figures 3A–F, administration of ICG-001 did not change the body weight, blood glucose, serum levels of triglyceride and total cholesterol and lipid deposition in the liver and kidney. However, renal dysfunction and tubular damage were clearly ameliorated by ICG-001, as reflected by serum creatinine level and renal expression of Kim-1 and NGAL (Figures 3G–I). In addition, ICG-001 also reduced the mRNA expression of a panel of proinflammatory cytokines in the kidney of mice fed with HFD (Figure 3J).

**Figure 4:**
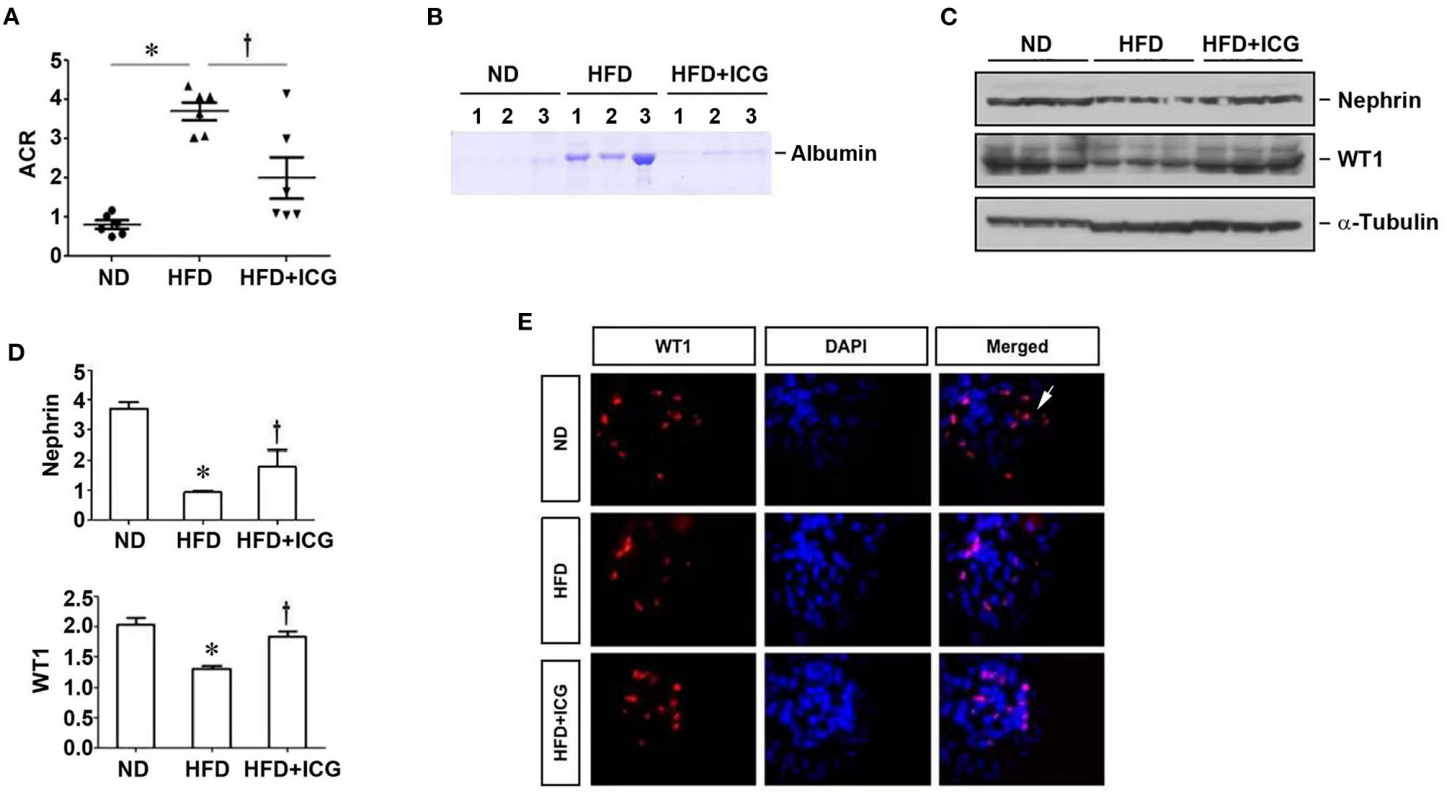
ICG-001 alleviates podocyte injury in mice fed with high-fat diet. Mice were given high-fat diet and administered with ICG-001 or vehicle at 4 weeks after high-fat diet. The experiments were terminated at 13 weeks. **(A)** ICG-001 reduced albuminuria. Urinary albumin to creatinine ratio (ACR) was presented. **(B)** Urinary proteins were analyzed on SDS-PAGE. **(C,D)** Kidney lysates were subjected to Western blot analyses of nephrin and WT1. Representative western blot **(C)** and quantitative data **(D)** are shown. **(E)** Immunofluorescence staining for WT1. ND, normal diet; HFD, high-fat diet; ICG, ICG-001. *P < 0.05, ND vs. HFD, *P* < 0.05 HFD vs. HFD + ICG, *n* = 6.

We found that blockade of Wnt/β-catenin signaling by ICG-001 also reduced proteinuria in mice fed with HFD (Figures 4A,B). ICG-001 restored podocyte-specific proteins nephrin and WT1 (Figures 4C–E). These results suggest that inhibition of Wnt/β-catenin signaling ameliorates HFD-induced renal lesions without affecting lipid deposition and metabolic profile.

### ICG-001 Ameliorates Palmitate-Triggered Podocyte Injury *in vitro*

**Figure 5:**
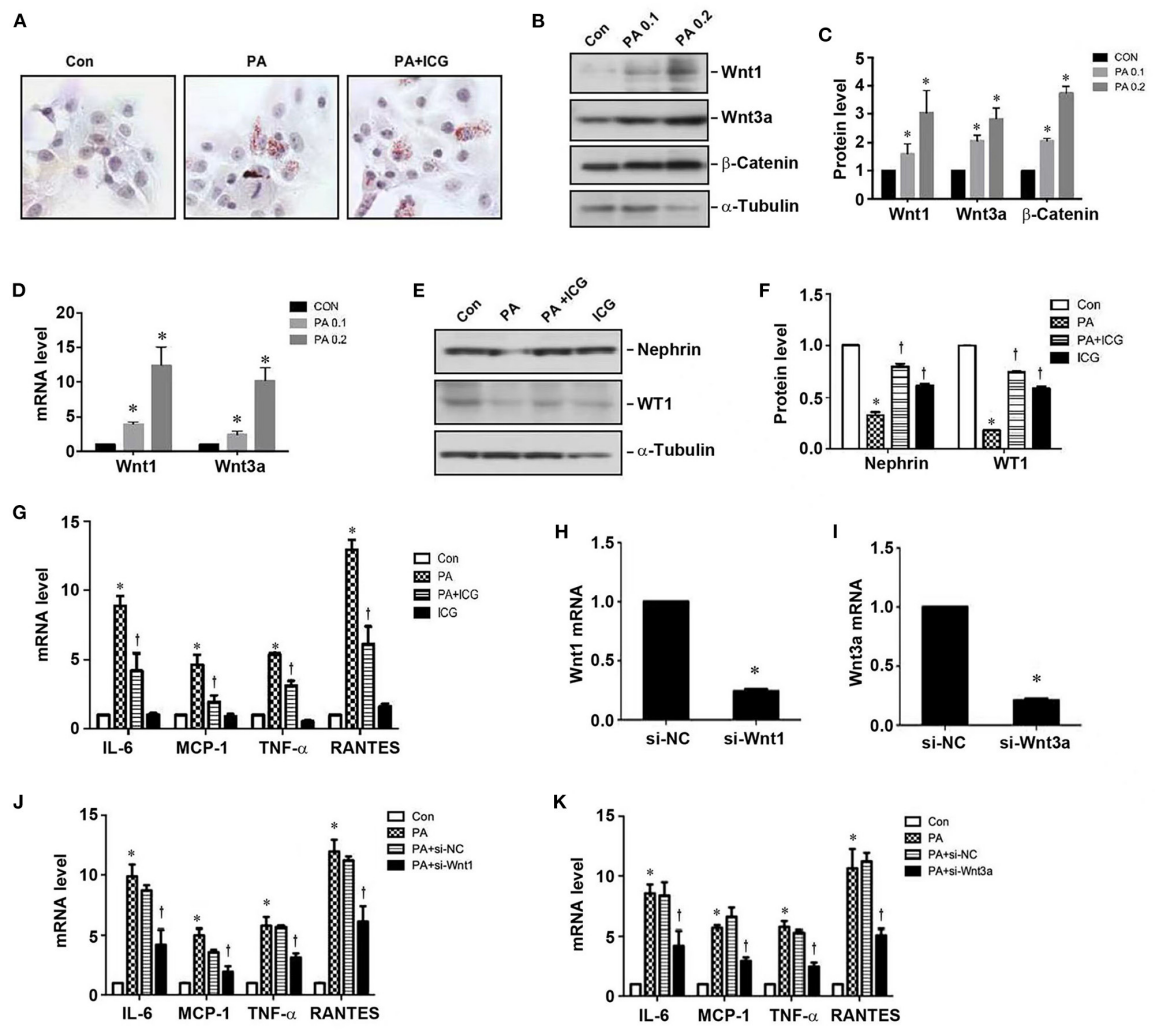
Inhibition of Wnt/β-catenin signaling ameliorates palmitate-induced podocyte injury. **(A)** Palmitate induced cellular lipid deposition in cultured podocytes, as shown by oil red staining. **(B,C)** Wetsern blotting show that palmitate induced protein expression of Wnt1, Wnt3a, and β-catenin in podocytes. Representative western blot **(B)** and quantitative data **(C)** are shown. **(D)** qRT-PCR shows that palmitate induced mRNA expression of Wnt1 and Wnt3a in podocytes. **(E,F)** Western blot analyses show that ICG-001 abolished PA-triggered loss of nephrin and WT1 in podocytes. **(G)** qRT-PCR shows that PA induced the mRNA expression of pro-inflammatory cytokines in podocytes, which was inhibited by ICG-001. **(H,I)** Knockdown of Wnt1 **(H)** and Wnt3a **(I)** expression by siRNA in podocytes. **(J,K)** qRT-PCR demonstrates that knockdown of either Wnt1 or Wnt3a inhibited the expression of various cytokines as indicated. CON, control; PA, palmitate; PA0.1: 0.1 mmol/L palmitate; PA0.2, 0.2 mmol/L palmitate; ICG, ICG-001. *P < 0.05, CON vs. PA, *P* < 0.05 PA vs. PA + ICG or PA+si-Wnt1/3a, *n* = 3.

To further study the role of Wnt/β-catenin in mediating HFD-induced kidney injury, we utilized mouse podocytes as an *in vitro* model system. To this end, mouse podocytes (MPC) were treated with palmitic acid (PA), a 16-carbon saturated fatty acid. As shown in Figure 5A, PA induced intracellular lipid deposition, which was not affected by ICG-001. PA induced Wnt1 and Wnt3a protein and mRNA expression in podocytes in a dose-dependent fashion (Figures 5B–D). Interestingly, blockade of Wnt/β-catenin signaling by ICG-001 restored nephrin and WT1 expression in podocytes (Figures 5E,F). ICG-001 also inhibited the mRNA expression of various cytokines including IL-6, MCP-1, TNF-α and RANTES in podocytes (Figure 5G). Furthermore, knockdown of Wnt1 or Wnt3a expression by siRNA also abolished PA-induced expression of various proinflammatory cytokines in podocytes (Figures 5H–K). These results suggest that Wnt/β-caenin signaling plays a critical role in mediating PA-induced podocytes injury and inflammatory responses *in vitro*, which is independent of affecting lipid deposition.

### AMPK Is a Downstream Mediator of Wnt Signaling

**Figure 6:**
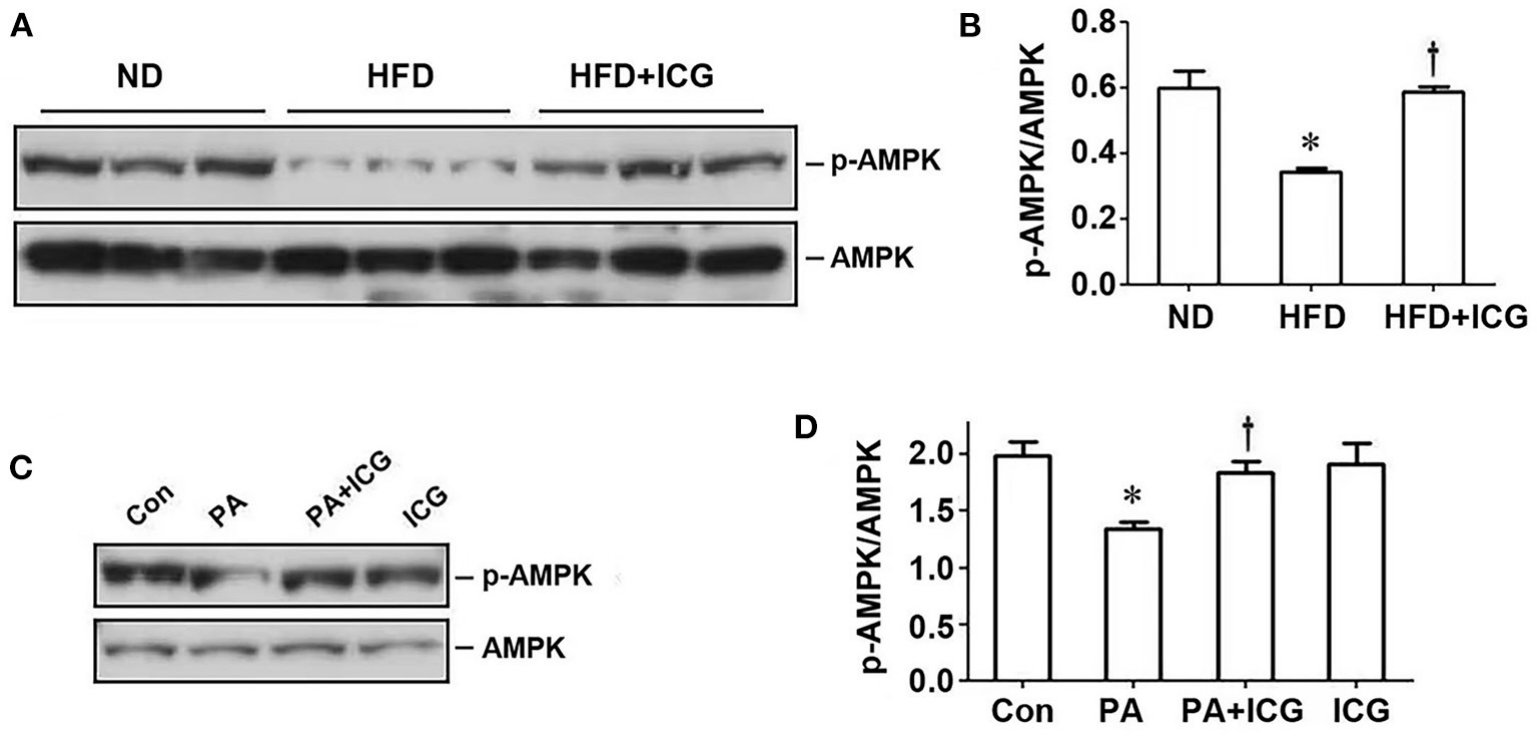
ICG-001 restores AMPK phosphorylation repressed by HFD *in vivo* or palmitate *in vitro*. **(A,B)** Western blot analyses show that ICG-001 restored renal AMPK phosphorylation in mice fed with high-fat diet for 13 weeks. Representative western blot **(A)** and quantitative data **(B)** are presented. **(C,D)** ICG-001 restored p-AMPK expression after palmitate treatment. Representative western blot **(C)** and quantitative data **(D)** are presented. Con, control; PA, palmitate; ICG, ICG-001. *P < 0.05, Con vs. PA, *P* < 0.05 PA vs. PA + ICG, *n* = 3.

AMP-activated protein kinase (AMPK) is a central regulator of energy homeostasis and metabolism. Therefore, we further examined the potential involvement of AMPK in HFD-induced kidney disease. As shown in Figures 6A,B, HFD suppressed AMPK phosphorylation in the kidney, which was largely restored by ICG-001. Similarly, in cultured podocytes, ICG-001 also restored the phosphorylated AMPK expression after PA treatment (Figures 6C,D). These studies imply that AMPK may be a downstream regulator of Wnt/β-catenin signaling in the pathogenesis of obesity-associated nephropathy.

## Discussion

Obesity-related renal injury could develop in the early course of obesity, which clinically manifests as proteinuria, glomerular hypertrophy, or obesity-associated focal segmental glomerulosclerosis. The present study demonstrates a series of metabolic alterations in HFD mice, including weight gain, elevated blood glucose, and accumulation of lipid droplets in the liver and kidney. Meanwhile, HFD induces a series of pathological changes in the kidney including cytokine expression, inflammation, tubular injury and proteinuria, leading to renal dysfunction. Our findings are in line with other reports showing that lipid accumulation causes structural and functional changes in podocytes, mesangial cells, and renal tubular epithelial cells (18). Most importantly, we show for the first time that Wnt/β-catenin signaling plays a pivotal role in mediating obesity-associated kidney injury.

Wnt proteins are a family of secreted lipid-modified glycoproteins with established roles in embryonic development and tissue homeostasis (19). The Wnt family contains 19 members in humans (20). Defects in Wnt signaling are associated with a variety of human diseases, ranging from developmental abnormalities, tissue fibrosis to cancer formation. Wnt/β-catenin signaling in adult kidneys is relatively silenced, but is up-regulated in virtually every experimental animal model, arranging from AKI to different forms of CKD (10). Reactivation of Wnt/β-catenin underscores the possible critical role of this developmental signal in subsequent repair or disease development after various injuries. Our data show that many Wnt ligands, especially Wnt1 and Wnt3a, are up-regulated in the kidney of mice fed with HFD. In addition, the expression of β-catenin, the principal downstream mediator of Wnt signaling, is also induced, indicating that high fat activates Wnt signaling. It is interesting to point out that Wnt proteins are often associated with lipid vesicles, which prolongs their activity (21). The lipidation of Wnt proteins has an imperative impact on their function. Local deposition of lipids in the kidney after HFD may promote the stability and function of Wnts in the kidney, aside from their upregulation. These effects may work in concert to promote the biological activity of Wnt proteins.

The present study explores the role of Wnt/β-catenin in obesity-associated nephropathy by inhibiting its signal transduction. A pharmacological inhibitor of Wnt signaling, ICG-001 that disrupts the interaction of β-catenin with cAMP response element binding protein (CREB)-binding protein (CBP) (22), is chosen for the loss-of-function analysis. With ICG-001 intervention, expression of tubular injury markers (Kim-1 and NGAL) and inflammatory cytokines is abolished and renal function restored, without affecting metabolic profile. At the same time, ICG-001 also attenuates proteinuria and ameliorates podocyte injury. It should be noted that proteinuria is often a result of an impaired glomerular filtration due to podocytes injuries. Therefore, proteinuria in HFD mice may reflect a direct damage to podocytes, and this notion is confirmed by the observation that key podocyte-specific proteins such as nephrin and WT1 are down-regulated in HFD mice. For modeling the impact of HFD, we choose cultured podocytes as an *in vitro* system. Similarly, exposing podocytes to palmitate results in increased intracellular lipid accumulation, accompanied by up-regulation of Wnt1, Wnt3a and β-catenin and loss of nephrin and WT1. These lesions in podocytes can be ameliorated by ICG-001, suggesting a pivotal role of Wnt/β-catenin signaling in mediating podocyte injury.

Alterations in lipid metabolism and deposition have also been reported in obese people with CKD (23). The accumulation of intracellular lipids is usually regulated by the balance between the influx, synthesis, and breakdown of fatty acids and oxidation and efflux (23, 24). Earlier studies show that obesity-related kidney damage is associated with decreased activity of AMPK in the kidney (24, 25). AMPK, expressed in the kidney abundantly, maintains energy balance via promoting energy-production pathways such as fatty acid oxidation and inhibiting fatty acid synthesis. Our findings are in accordance with this notion. Reduction of phosphorylated AMPK was observed in both HFD kidney and palmitate-treated podocytes, and ICG-001 directly restores phosphorylated AMPK. Growing evidence suggests that high-fat diet-induced kidney injury is intricately linked to AMPK activity and its downstream signaling (26, 27). AMPK can be activated via phosphorylation by upstream kinases (28). Activation of AMPK normalizes renal lipid content and ameliorates obesity-associated renal injury through improving lipid metabolism, mitochondrial homeostasis, oxidative stress, autophagy, inflammation and fibrosis (29–31). The fact that inhibition of Wnt/β-catenin by ICG-001 restores phosphorylated AMPK suggests that this signaling targets a major pathway in connecting metabolic disorder to obesity-associated nephropathy.

In summary, we have demonstrated that Wnt signaling, especially Wnt1 and Wnt3a, plays a pivotal role in mediating HFD-induced kidney injury. Therefore, targeting this signaling by ICG-001 or other strategies may be a potential remedy for treating obesity-associated kidney disease.

## Data Availability Statement

The original contributions presented in the study are included in the article/Supplementary Material, further inquiries can be directed to the corresponding authors.

## Ethics Statement

The animal study was reviewed and approved by the Institutional Animal Care and Use Committee at the University of Pittsburgh.

## Author Contributions

YY, HM, and HZ performed experiments. YY prepared figures and drafted manuscript. YL contributed to the conception and design of the study. CY edited and revised manuscript. All authors approved final version of manuscript.

## Funding

This work was supported by National Institutes of Health Grant DK064005 and National Natural Science Foundation of China (81920108007, 82170696, and 81600523).

## Conflict of Interest

The authors declare that the research was conducted in the absence of any commercial or financial relationships that could be construed as a potential conflict of interest.

## Publisher's Note

All claims expressed in this article are solely those of the authors and do not necessarily represent those of their affiliated organizations, or those of the publisher, the editors and the reviewers. Any product that may be evaluated in this article, or claim that may be made by its manufacturer, is not guaranteed or endorsed by the publisher.
