# Supplementary material for: High Fat Diet Induces Kidney Injury via Stimulating Wnt/β-Catenin Signaling
Source: Front Med (Lausanne). 2022 Apr 7;9:851618. doi: 10.3389/fmed.2022.851618 (PMC9021428; doi:10.3389/fmed.2022.851618)
Supplement: Supplementary file 1 [file Table_1.DOCX]

|  | Forward Primer(5'-3') | Reverse Primer(5'-3') |
| --- | --- | --- |
| KIM-1 | ACATATCGTGGAATCACAACGAC | ACTGCTCTTCTGATAGGTGACA |
| NGAL | CAGTGCCGCCGATTACTACTT | AGGAGGCTAATAGTTTGTCGGAT |
| MCP-1 | CCCACTCACCTGCTGCTAC | TTCTTGGGGTCAGCACAGA |
| TNF-α | CGTAGCAAACCACCAAGTG | CCTTGAAGAGAACCTGGGAG |
| IL-6 | GGCGGATCGGATGTTGTGAT | GGACCCCAGACAATCGGTTG |
| RANTES | TTTGCCTACCTCTCCCTCG | CGACTGCAAGATTGGAGCACT |
| IL-1β | GAAATGCCACCTTTTGACAGTG | TGGATGCTCTCATCAGGACAG |
| Arg | GGGAGAGCAAACGGAACCG | CTGCCGAATTTTTCGCTGTCG |
| Wnt1 | ATCCTGCACCTGCGACTACAG | GGCGACTTCTCGAAGTAGACC |
| Wnt2 | ATCCAAAGAAGAAAGGAAGT | TTTACACTCACACTTGGTCA |
| Wnt3a | CTCCTCTCGGATACCTCTTAGTG | CCAAGGACCACCAGATCGG |
| Wnt4 | AAGAGGAGACGTGCGAGAAAC | GTCCCTTGTGTCACCACCTT |
| Wnt5a | AATATTAAGCCCAGGAGTGG | TGGCAGAGTTTCTTCTGTCCT |
| Wnt5b | CATTGGGATGGGTTGAGG | CAGGAAGTTGGCAGCACAC |
| Wnt7 | CCTGGGCCACCTCTTTCT | TGGGAGCCAGGCCTGGGAT |
| Wnt9b | ACCTGAAGCAGTGTGACCTAC | GCTCCTGCCTGAACTGGAA |
| Wnt10a | CAGATCGCCATCCATGAGTG | ACCGCAAGCCTTCAGTTTACC |
| Wnt10b | GCGGGTCTCCTGTTCTTGG | CCGGGAAGTTTAAGGCCCAG |
| Wnt11 | GCACTGAATCAGACGCAACAC | CGACAGGGCATACACGAAGG |
| β-actin | CACCCGCGAGTACAACCTTC | CCCATACCCACCATCACACC |
